# Betaine and I-LG may have a predictive value for ATB: A causal study in a large European population

**DOI:** 10.1371/journal.pone.0306752

**Published:** 2024-07-05

**Authors:** Xiaomin Xian, Li Li, Jing Ye, Wenxiu Mo, Dabin Liang, Minying Huang, Yue Chang, Zhezhe Cui

**Affiliations:** 1 School of Public Health, the Key Laboratory of Environmental Pollution Monitoring and Disease Control, Ministry of Education, Guizhou Medical University, Guiyang, Guizhou, China; 2 Department of Dermatology and Venereology, The First Affiliated Hospital of Guangxi Medical University, Nanning, Guangxi, China; 3 Guangxi Key Laboratory of Major Infectious Disease Prevention and Control and Biosafety Emergency Response, Guangxi Key Discipline Platform of Tuberculosis Control, Guangxi Centre for Disease Control and Prevention, Nanning, Guangxi, China; 4 School of Public Health and Management, Youjiang Medical University for Nationalities, Baise, Guangxi, China; 5 School of Medicine and Health Management, Guizhou Medical University, Guiyang, Guizhou, China; UT Health San Antonio: The University of Texas Health Science Center at San Antonio, UNITED STATES

## Abstract

**Purpose:**

To analyze the causal relationship between 486 human serum metabolites and the active tuberculosis (ATB) in European population.

**Methods:**

In this study, the causal relationship between human serum metabolites and the ATB was analyzed by integrating the genome-wide association study (GWAS). The 486 human serum metabolites were used as the exposure variable, three different ATB GWAS databases in the European population were set as outcome variables, and single nucleotide polymorphisms were used as instrumental variables for Mendelian Randomization. The inverse variance weighting was estimated causality, the MR-Egger intercept to estimate horizontal pleiotropy, and the combined effects of metabolites were also considered in the meta-analysis. Furthermore, the web-based MetaboAnalyst 6.0 was engaged for enrichment pathway analysis, while R (version 4.3.2) software and Review Manager 5.3 were employed for statistical analysis.

**Results:**

A total of 21, 17, and 19 metabolites strongly associated with ATB were found in the three databases after preliminary screening (P < 0.05). The intersecting metabolites across these databases included tryptophan, betaine, 1-linoleoylglycerol (1-monolinolein) (1-LG), 1-eicosatrienoylglycerophosphocholine, and oleoylcarnitine. Among them, betaine (*I*^2^ = 24%, *P* = 0.27) and 1-LG (*I*^2^ = 0%, *P* = 0.62) showed the lowest heterogeneity among the different ATB databases. In addition, the metabolic pathways of phosphatidylethanolamine biosynthesis (*P* = 0.0068), methionine metabolism (*P* = 0.0089), betaine metabolism (*P* = 0.0205) and oxidation of branched-chain fatty acids (*P* = 0.0309) were also associated with ATB.

**Conclusion:**

Betaine and 1-LG may be biomarkers or auxiliary diagnostic tools for ATB. They may provide new guidance for medical practice in the early diagnosis and surveillance of ATB. In addition, by interfering with phosphatidylethanolamine biosynthesis, methionine metabolism, betaine metabolism, oxidation of branched-chain fatty acids, and other pathways, it is helpful to develop new anti-tuberculosis drugs and explore the virulence or pathogenesis of ATB at a deeper level, providing an effective reference for future studies.

## Introduction

It was estimated that a quarter of the global population is in a state of latent infection with *Mycobacterium tuberculosis (M*. *tb)*, which is at risk of developing active tuberculosis (ATB) disease when host immune regulation is out of balance [1, 2]. While about 90–95% of people infected with *M*. *tb* do not develop ATB and remain asymptomatic [3]. Once developed into ATB, it most often involves the lungs, but also easily causes lymphadenopathy, skin diseases, neurological deficits, or other disseminated diseases, which not only causes serious damage to human health but also increases the economic burden and psychological burden of patients. According to the Global TB Report 2023, about 1.3 million people will die from ATB worldwide in 2022 [4]. Although great progress has been made in the prevention and control of ATB, it remains a public health problem worldwide.

In addition to environmental, pathogen, and socioeconomic factors [5, 6], host genetic diversity plays a significant role in the development of ATB [7]. At the same time, emerging metabolomics has been widely used to identify potential diagnostic biomarkers for various diseases, including ATB [8, 9]. Excepting diagnosis, these metabolites can also be used as biomarkers to elucidate disease mechanisms, as they can truly reflect the adaptation of bacteria to the metabolome due to growth in vivo and host response to infection and disease [10]. Studies have shown that inosine can be used as a marker of latent infection of tuberculosis, and metabolites such as betaine, branch-chain amino acids, and serum alanine have important effects on the pathogenesis of TB [11]. Similarly, tuberculous stearic acid (TBSA), branched-chain fatty acids, and vitamin D are also frequently studied as metabolites of ATB related [7, 12]. These metabolites may participate in the metabolic pathways of amino acids and fatty acids, and the abnormal metabolism of these metabolites may lead to the disorder of pro-inflammatory cytokine response and the change of anti-inflammatory response, leading to the decline of immunity, and then promote the accelerated proliferation of bacteria and the spread of infectious lesions.

However, the majority of these reports were derived from cross-sectional observational studies, which limits the strength of causal inference. Therefore, the present study collected serum metabolomic data from a comprehensive European population and utilized metabolism-associated single nucleotide polymorphisms (SNPs) as instrumental variables (IVs) in mendelian randomization (MR) validation in genomics to assess the causal influence of genetic replacement elements in metabolic pathways and the development of ATB, thereby providing new insights into the underlying mechanisms of ATB. Therefore, this study included three different European ATB GWAS datasets to comprehensively analyze the influence of serum metabolites on ATB pathogenesis. This study is of great significance for the monitoring and diagnosis of ATB in the future.

## Methods

### Study design

**Fig 1:**
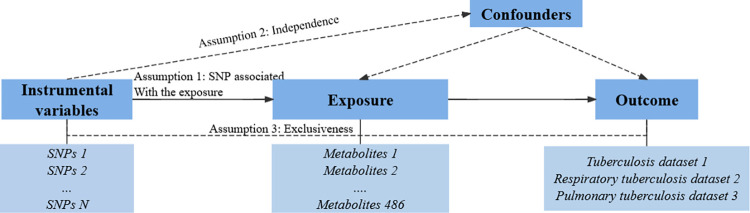
Schematic representation of MR analysis. (We selected significant IVs for 486 metabolites and ATB and explored causality. Acyclic graphs are used to illustrate the three basic assumptions of the MR Analysis).

In this study, we comprehensively evaluated the relationship between 486 human serum metabolites and ATB based on a rigorous MR design. At its core, MR used genetic data as IVs to explore causal associations between a given exposure and outcome. A scientific MR study must include the testing of three hypotheses [13]: 1) the association hypothesis: genetic IVs are strongly associated with the study’s exposure; 2) the independence hypothesis: genetic IVs are not associated with outcome and are independent of any confounding factors, known or unknown; 3) the exclusive hypothesis: IVs can only affect the outcome through exposure factors. Therefore, the association obtained by MR is not affected by causal inversion and is not affected by confounding factors. In addition, the advantages of MR include the small measurement error of genetic variation and its effects, and the availability of publicly available GWAS data. However, MR can also have disadvantages, on the one hand, MR studies may be error-prone if the genetic variation is pleiotropy, and on the other hand, there may be linkage disequilibrium reactions (LD), since not all genes determining the trait are independently and randomly isolated. So, this study will set some constraints to reduce the consequences of LD. A schematic of this two-sample MR study is shown in Fig 1. At the same time, we used the STROBE-MR checklist to ensure the reliability and scientific of the study [14] (S1 Table).

### Data sources

#### GWAS data of human serum metabolites (exposure)

Shin et al. [15] obtained genome-wide association aggregate datasets for 486 human serum metabolites in this study. The service platform is a collection of relatively complete European human serum metabolomics data and can be from the GWAS server publicly available (https://gwas.mrcieu.ac.uk/). Developed by the Integrated Epidemiology Unit (IEU) at the University of Bristol, this resource is a manually curated collection of the complete GWAS summary datasets and is available for download as an open-source file or by querying the complete database online. A total of 7,824 participants for the GWAS of human serum metabolites were recruited from two European population cohorts, including 1,768 participants from the KORA and 6,056 from the TwinUK, and approximately 2.1 million SNPs were included in the study. Out of the 486 metabolites, 309 have been named, according to the Kyoto Encyclopedia of Genes and Genomes (KEGG) database definition of genomes [16]. They can be divided into 8 Super-pathways (Amino acids, Carbohydrates, Cofactors and Vitamins, Energy, Lipids, Nucleotides, Peptides, and Xenobiotics) for integration and interpretation of large molecular datasets. The chemical properties of an additional 177 unknown metabolites have not been fully determined.

#### GWAS data for ATB (outcome)

In this study, we will use three different ATB databases to match 486 human serum metabolites and screen out the SNPs with genetic significance. To avoid racial differences, metabolites, and ATB genetic information were selected from GWAS datasets of the European population. The different ATB GWAS pooled data from the ieu open GWAS project were finn-b-AB1_TUBERCULOSIS database, finn-b-TBC_RESP database, and ebi-a-GCST90018892 database. These inclusion criteria for the cohort were based on the diagnostic protocol of ATB [17]. The GWAS pooled data included a total of 2,937 cases and 911,722 controls, with 56.95 million SNPs included in the analysis. We used SNPs associations of 486 human serum metabolites with ATB and their significant variables to explore the causal relationship.

### Selecting of IVs

The selection of IVs in this MR Analysis were based on three basic premises. First, for each metabolite, we set *P* < 1×10^−5^ as a genome-wide significance threshold to select strongly correlated SNPs [18]. Second, a clustering program was implemented using R software to identify independent variants. Within a range of 10,000 kilobases (kb), R^2^ ≤ 0.01 were used to indicate LD, and identify independent SNPs [19]. Finally, to quantitatively verify whether the selected SNPs were powerful tools, the *F* statistic for each metabolite was calculated in this study. In general, an *F* ≥ 10 threshold was adopted for the following analyses. Moreover, considering the influence of alleles, we performed direct deletion of SNP palindromes [20]. MR of polygenic variants were performed using the pooled data.

### MR analysis

The standard inverse variance weighted (IVW) method was employed to assess the causal association between metabolites and ATB. When all three main assumptions of IVs are met, the IVW method can provide a more precise estimate of the causal effect of exposure and is considered as the approach for robust causal detection [15]. If the null hypothesis were rejected, indicating that one or more variants may be multivariable, a random effects IVW was performed instead of a fixed effects IVW [21]. Conversely, if some IVs do not conform to the hypothesis, the analysis may gave inaccurate results. Therefore, we performed the following sensitivity analysis: 1) Q tests were performed on both IVW and MR-Egger methods to detect potential violation of heterogeneity assumption among individual IVs. Cochran’s Q test, commonly used in studies assessing diagnostic accuracy heterogeneity, was utilized [22]; 2) MR-Egger intercept was estimated to evaluate horizontal pleiotropy and ensure that genetic variants were independently associated with both metabolites and ATB outcomes [23]. The MR-Egger method was employed for interpreting the results obtained from MR analysis; 3) Additional analyses such as weighted median (WM) and weighted mode were implemented to enhance reliability and stability in hypothesis testing; 4) Single SNP analysis along with leave-one-out test were conducted to assess the plausibility of observed associations attributed to individual SNP.

### Meta-analysis

If the results obtained from different databases were controversial, to ensure more convincing results, we conducted heterogeneity tests on these metabolites. Review Management 5.3 software was engaged to conduct a meta-analysis of the individual human serum metabolites that were identified as having a causal relationship with ATB and examined the overall estimated effect size. In this study, we used the odds ratio (*OR*) as the scale indicator to estimate the unknown data in the exposure group, and finally presented the results through forest plots. When *I*^*2*^ ≥ 50% or *P* < 0.05, high heterogeneity was indicated, and a random effects model should be used; when *I*^*2*^ < 50% and *P* ≥ 0.05, a fixed effects model should be used.

### Enrichment analysis

MetaboAnalyst 6.0, a web-based tool for analyzing metabolomics data, was utilized to perform metabolic pathway analysis, interpretation, and integration with other omics data (https://www.metaboanalyst.ca/) [24]. The pathway and enrichment analysis module were applied to screen out metabolite clusters or super pathways that may be associated with metabolic processes and analyze their potential associations with ATB. Refer to the Small Molecule Pathway Database (SMPDB) and the Kyoto Encyclopedia of Genes and Genomes (KEGG) database. The significance level of pathways was less than 0.05.

### Statistical analysis

All MR Analyses were performed using the "TwoSampleMR" package in R (version 4.3.2). Both the volcano and veen plots were drawn using Bioladder (https://www.bioladder.cn/), and the heat plots were drawn using Chiplot (https://www.chiplot.online/). The *P* < 0.05 of the IVW, since the threshold of the *P* value was artificially specified, even the smallest *P* value will give false positive results. Therefore, to reduce the chance of making Class I errors, we needed to perform multiple tests to eliminate false positives by correcting the threshold of *P*-values by Bonferroni, ensuring that the metabolites selected were diagnostically significant. The correction formula is P* (1/N), where "P" is the original threshold and "N" is the total number of tests [15]. Similarly, Bonferroni-*P* < 0.05 was considered statistically significant. The *OR* is used to estimate the size and direction of the metabolite effect and its corresponding 95% confidence interval (*CI*).

### Ethics statement

The requirement for obtaining informed consent was waived in this study due to the utilization of publicly available data sets within the database, which does not encompass any medical records pertaining to research subjects and can be accessed by anyone through the website.

## Results

### Selection of genetic IVs for the three GWAS

In this study, a total of 486 serum metabolites data were matched to three the ATBGWAS online databases, and the selection of strongly associated SNPs was based on a genome-wide significance threshold (*P* < 1×10^−5^). Unfortunately, we were unable to identify relevant human serum metabolites in our reverse causal assessment. Consequently, different databases yielded varying numbers of associated metabolites based on the forward MR Study. Consequently, different databases yielded varying numbers of related metabolites. Among them, 485 metabolites were selected for both finn-b-AB1_TUBERCULOSIS and finn-b-TBC_RESP, and 486 metabolites were selected for ebi-a-GCST90018892. However, metabolites that passed the IVW test and Bonferroni P < 0.05 were found in 21 in finn-b-AB1_TUBERCULOSIS, 17 in finn-b-TBC_RESP, and 19 in ebi-a-GCST90018892. These metabolites were involved in seven super-pathways. In comparison, Lipid was the highest constituent, followed by Amino acids and Xenobiotics, and Carbohydrates and Nucleotides were the lowest (S2 Table). Finally, the IVs of finn-b-AB1_TUBERCULOSIS database contained a total of 2346 SNPs, with a median of 20 SNPs. The IVs of finn-b-TBC_RESP database contain 400 SNPs, and the median SNPs was 15. Meanwhile, the IVs of ebi-a-GCST90018892 database contain only 18,364 SNPs, and the median was 18. All three databases had *F* > 10, indicating that weak tool bias was unlikely to be significant (Table 1).

**Table 1:** Results of SNPs associations with 486 human serum metabolites from three GWAS databases.

	finn-b-AB1_TUBERCULOSIS	finn-b-TBC_RESP	ebi-a-GCST90018892
Number of case	1193	849	895
Number of control	217599	217632	476491
Total number of SNPs	16380466	16380466	24189689
Number of IVs	21	17	19
SNPs of IVs	2346	400	18364
Median SNPs of IVs	20	15	18
Unknown metabolites	7	8	7
Known metabolites	14	9	12
Amino acid	5	4	3
Carbohydrate	1	-	-
Cofactors and vitamins	1	-	1
Energy	-	-	-
Lipid	5	3	6
Nucleotide	1	-	-
Peptide	1	1	-
Xenobiotics	-	1	2

#### Impact of metabolites on ATB

**Fig 2:**
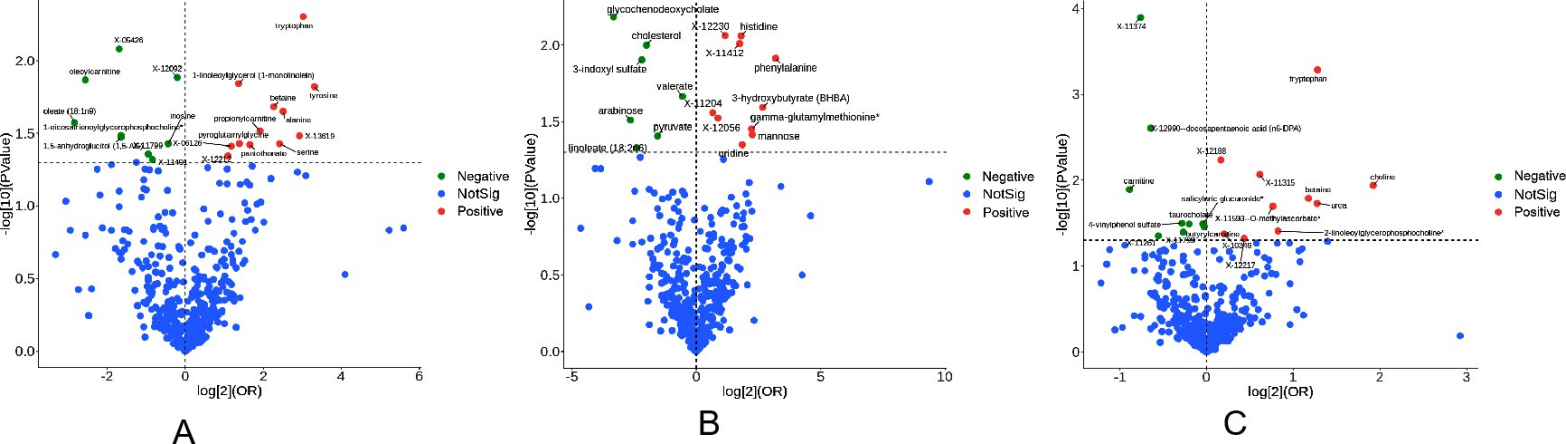
Volcano plot showing the impact of metabolites on TB. (The plot includes the *OR* on a log2 scale and the negative log10 scale of the *P*-values, which are estimated using the IVW method. A, finn-b-AB1_TUBERCULOSIS; B, finn-b-TBC_RESP; C, ebi-a-GCST90018892).

Metabolites were screened in three databases, and 57 human serum metabolites were found to be significantly associated with ATB (S3 Table). All metabolic analyses used random-effects IVW as the primary analysis method, with no evidence of heterogeneity and no weak tools. The preliminary analysis identified 21 metabolites significantly associated with ATB in finn-b-AB1_TUBERCULOSIS database, with 12 showing a positive association and 9 showing a negative association. In finn-b-TBC_RESP database, 17 metabolites were found to be statistically significant, 10 were positively associated with ATB, and 7 were negatively associated with ATB. There were 19 significant metabolites in ebi-a-GCST90018892 database, 10 were positively associated with ATB and 9 were negatively associated. A volcanic plot of human serum metabolites associated with ATB in the three databases is shown in Fig 2.

### Relationship between three intersection metabolites and ATB

**Fig 3:**
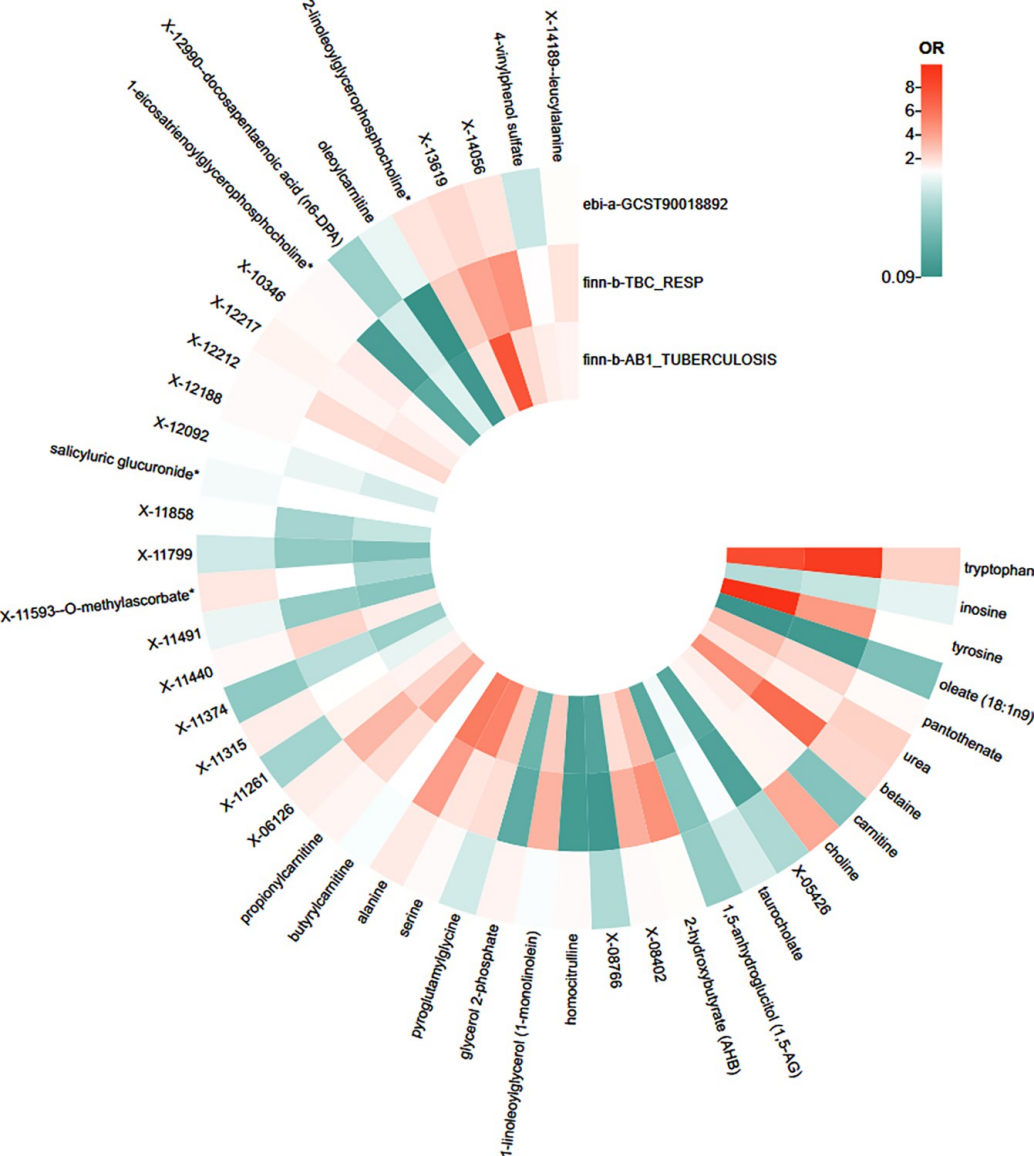
Heat plot of statistically significant metabolites in any database.

The heat plot shows the metabolites with statistical significance in any database and the corresponding *OR* values in different databases (Fig 3). The known metabolites that were positively correlated with ATB regardless of statistical significance of *P* values were tryptophan, tyrosine, pantothenate, urea, betaine, choline, 1-linoleoylglycerol (1-monolinolein) (1-LG), 2-hydroxybutyrate (AHB), serine, alanine, propionylcarnitine, and 2-linoleoylgly cerophosphocholine*. The negative correlations were included inosine, oleate (18:1n9), taurocholate, 1,5-anhydroglucitol (1,5-AG), X-12990—docosapentaenoic acid (n6-DPA), and oleoylcarnitine.

**Fig 4:**
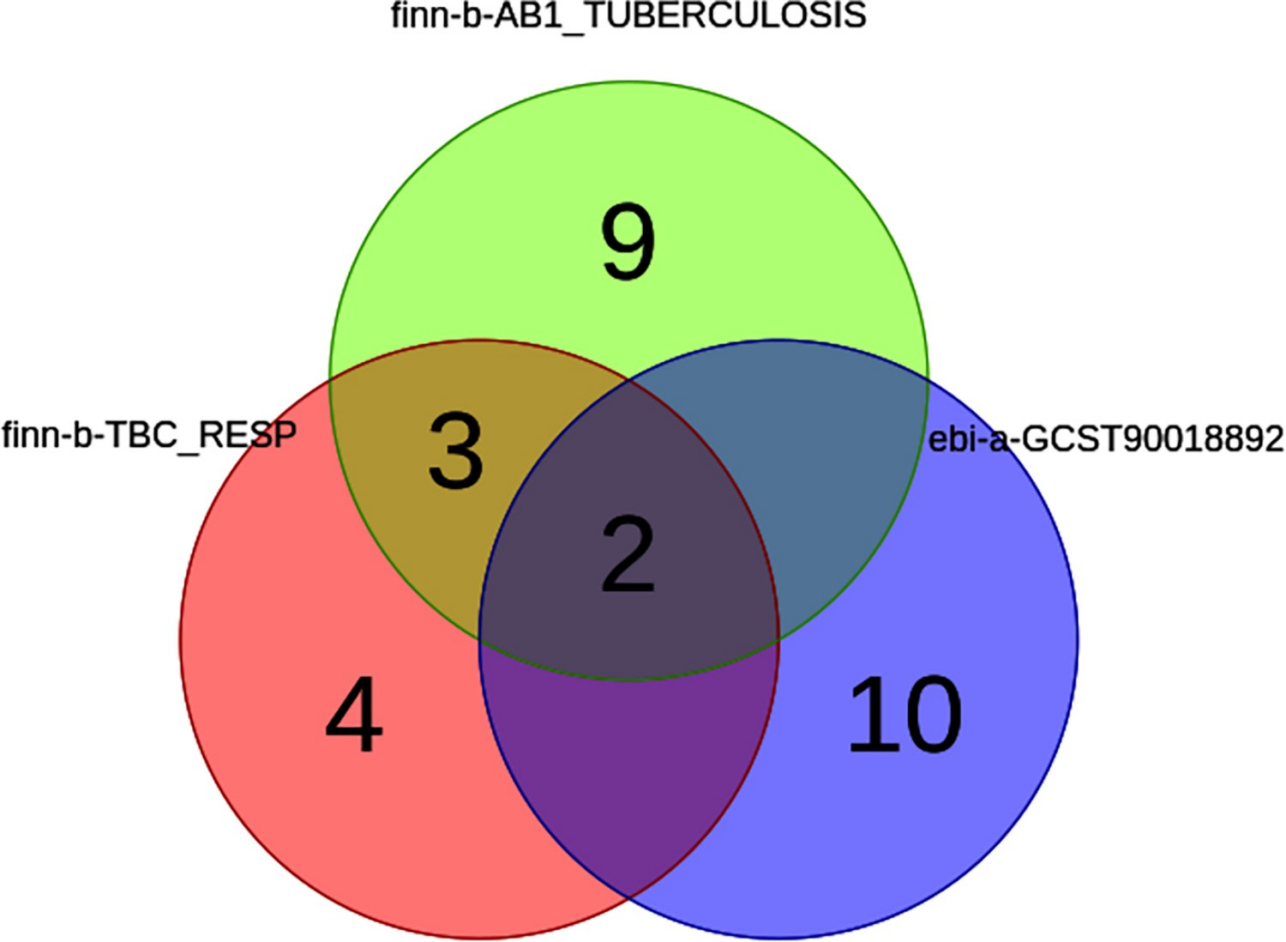
The Veen plot shows the intersecting metabolite numbers of different databases.

In addition to the 22 unknown metabolites, the other 35 known metabolites have different intersections between different databases (Fig 4). The specific metabolite names and related *OR* (95%*CI*) are shown in Tables 2 and 3. The significance of two metabolites, tryptophan and betaine, was observed across all three databases, indicating their super-pathway as amino acids. Both of these metabolites were positively correlated with ATB and were risk factors for ATB. The metabolites that were significant in finn-b-AB1_TUBERCULOSIS and finn-b-TBC_RESP databases included 1-LG, 1-eicosatrienoylgly cerophosphocholine, and oleoylcarnitine, in which 1-LG is positively correlated with ATB and has a promoting effect, while 1-eicosatrienoylcholine and oleoylcarnitine are negatively correlated with ATB and are protective factors.

**Table 2:** The specific names of intersecting metabolites.

finn-b-AB1_TUBERCULOSIS	finn-b-TBC_RESP	ebi-a-GCST90018892	Count	Values
TRUE	TRUE	TRUE	2	tryptophan | betaine
FALSE	FALSE	TRUE	10	urea | carnitine | choline | taurocholate | butyrylcarnitine | X-11593—O-methylascorbate | salicyluric glucuronide | X-12990—docosapentaenoic acid (n6-DPA) | 2-linoleoylgly cerophosphocholine | 4-vinylphenol sulfate
TRUE	TRUE	FALSE	3	1-linoleoylglycerol (1-monolinolein) | 1-eicosatrienoylgly cerophosphocholine | oleoylcarnitine
TRUE	FALSE	FALSE	9	inosine | tyrosine | oleate (18:1n9) | pantothenate | 1,5-anhydroglucitol (1, 5—AG) | pyroglutamylglycine | serine | alanine | propionylcarnitine
FALSE	TRUE	FALSE	4	2-hydroxybutyrate (AHB) | homocitrulline | glycerol 2-phosphate | X-14189—leucylalanine

**Table 3:** Significantly metabolites are present in at least two databases.

Metabolites	finn-b-AB1_TUBERCULOSIS		finn-b-TBC_RESP		ebi-a-GCST90018892	
	OR (95%CI)	*P* value	OR (95%CI)	*P* value	OR (95%CI)	*P* value
tryptophan	8.14 (1.88 35.17)	0.0050	9.12 (1.62–51.39)	0.0122	2.43 (1.47–4.01)	0.0005
betaine	4.82 (1.27 18.28)	0.0207	6.36 (1.25–32.28)	0.0255	2.26 (1.16–4.40)	0.0163
1-linoleoylglycerol (1-monolinolein)	2.58 (1.21 5.52)	0.0144	3.49 (1.37–8.89)	0.0087	-	-
1-eicosatrienoylgly cerophosphocholine	0.32 (0.11 0.91)	0.0327	0.22 (0.07–0.72)	0.0125	-	-
oleoylcarnitine	0.17 (0.04 0.69)	0.0135	0.10 (0.02–0.52)	0.0066	-	-

### Results of meta-analysis

**Fig 5:**
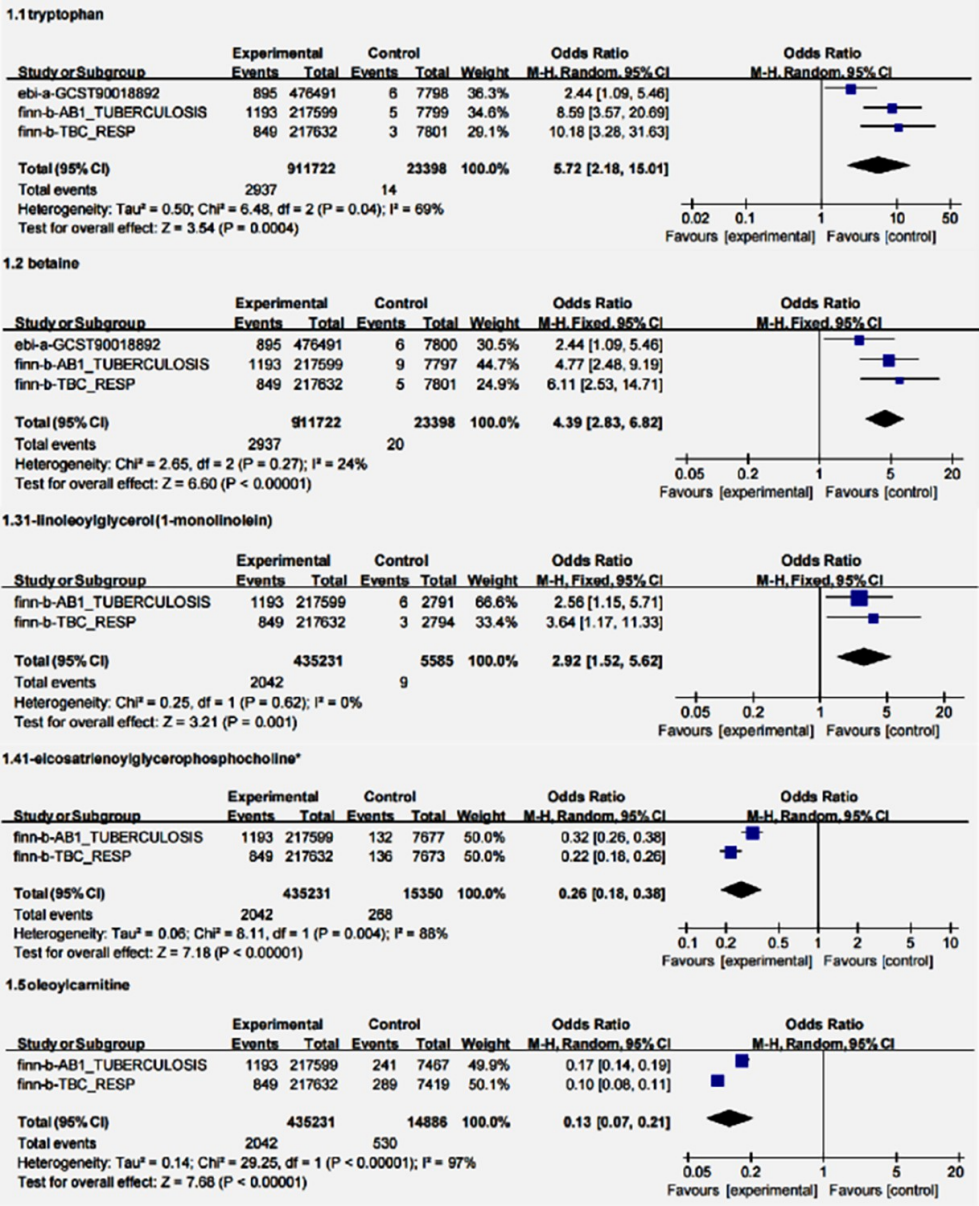
The combined effects of five metabolites are estimated in the forest plots.

As can be seen from Table 3, the *OR* values of metabolites in different databases were different. To explore the combined effect size of each metabolite, a meta-analysis was conducted. The results suggested low heterogeneity of betaine (*I*^*2*^ = 24%, *P* = 0.27), and even complete homogeneity of I-LG (*I*^*2*^ = 0%, *P* = 0.62). However, the final combined effect of the five metabolites was statistically significant (*P* < 0.05). Among them, tryptophan, betaine, and I-LG were risk factors, and 1-eicosatrienoylgly cerophosphocholine and oleoacylcarnitine were protective factors for the development of ATB (Fig 5).

### Enrichment analysis results of known metabolites

**Fig 6:**
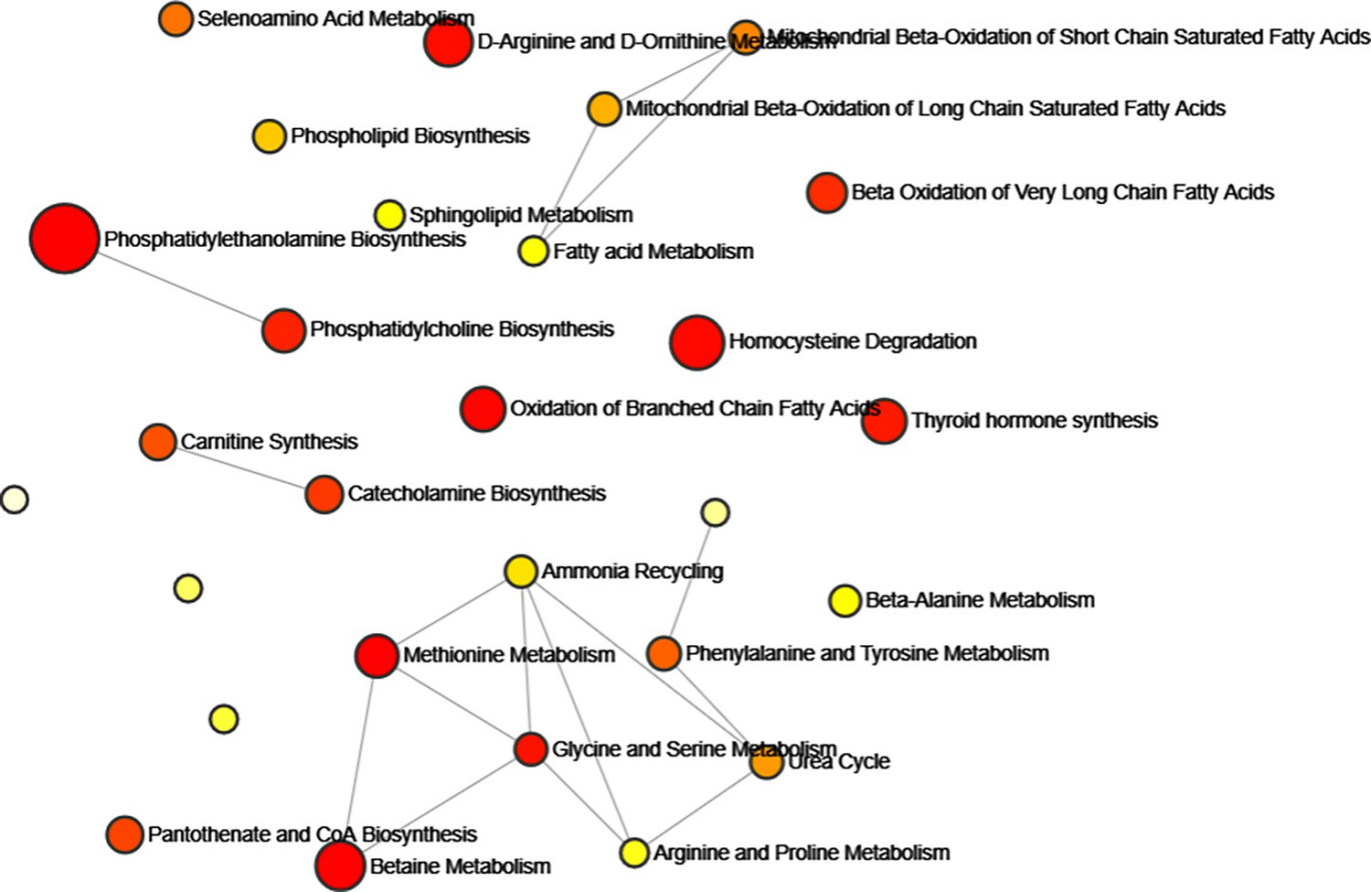
Metabolite enrichment pathway network for MR selection. (Colors range from light yellow to dark red, indicating the degree of enrichment significance, and the size of the circles reflects the degree of enrichment proportion).

35 known metabolites significantly associated with ATB were entered into the Metabolic Analyzer 6.0 platform to identify various potential metabolic pathways involved in the pathogenesis of ATB. Table 4 and Fig 6 demonstrate the interaction network among the metabolic pathways involved in this study. The metabolic pathways with high enrichment degree and proportion were the phosphatidylethanolamine (PE) biosynthesis (*P* = 0.0068), methionine metabolism (*P* = 0.0089), betaine metabolism (*P* = 0.0205) and oxidation of branched chain fatty acids (*P* = 0.0309).

**Table 4:** Metabolite enrichment pathways selected by MR.

Metabolite Set	Total	Expected	Hits	P value
Phosphatidylethanolamine Biosynthesis	12	0.132	2	0.0068
Methionine Metabolism	42	0.461	3	0.0089
Betaine Metabolism	21	0.231	2	0.0205
Oxidation of Branched Chain Fatty Acids	26	0.285	2	0.0309
Homocysteine Degradation	9	0.0988	1	0.0949
D-Arginine and D-Ornithine Metabolism	11	0.121	1	0.1150
Glycine and Serine Metabolism	59	0.648	2	0.1330
Thyroid hormone synthesis	13	0.143	1	0.1340
Phosphatidylcholine Biosynthesis	14	0.154	1	0.1440
Beta Oxidation of Very Long Chain Fatty Acids	17	0.187	1	0.1720
Catecholamine Biosynthesis	20	0.22	1	0.2000
Pantothenate and CoA Biosynthesis	21	0.231	1	0.2090
Carnitine Synthesis	22	0.242	1	0.2180
Phenylalanine and Tyrosine Metabolism	27	0.296	1	0.2610
Selenoamino Acid Metabolism	27	0.296	1	0.2610
Mitochondrial Beta-Oxidation of Short Chain Saturated Fatty Acids	27	0.296	1	0.2610
Urea Cycle	28	0.307	1	0.2690
Mitochondrial Beta-Oxidation of Long Chain Saturated Fatty Acids	28	0.307	1	0.2690
Phospholipid Biosynthesis	29	0.318	1	0.2770
Ammonia Recycling	31	0.34	1	0.2940
Beta-Alanine Metabolism	34	0.373	1	0.3170
Sphingolipid Metabolism	40	0.439	1	0.3630
Fatty acid Metabolism	43	0.472	1	0.3840
Arginine and Proline Metabolism	52	0.571	1	0.4450
Tryptophan Metabolism	59	0.648	1	0.4890
Bile Acid Biosynthesis	65	0.714	1	0.5240
Tyrosine Metabolism	70	0.768	1	0.5510
Purine Metabolism	73	0.801	1	0.5670

## Discussion

This study employed a two-sample MR model, utilizing genetic variants as IVs, to elucidate the causal relationship between human serum metabolites and ATB. Firstly, 15 known metabolites (tryptophan, tyrosine, pantothenate, pyroglutamylglycine, serine, alanine, propionylcarnitine, betaine, 2-hydroxybutyrate (AHB), 1-LG, X-14189—leucylalanine, urea, choline, X-11593—O-methylascorbate, and 2-linoleoylgly cerophosphocholine) were found to be risk factors for the development of ATB, while 13 known metabolites (inosine, oleate (18:1n9), 1,5-anhydroglucitol (1,5-AG), homocitrulline, glycerol 2-phosphate, 1-eicosatrienoylglycerophosphocholine, oleoylcarnitine, carnitine, taurocholate, butyrylcarnitine, salicyluric glucuronide, X-12990—docosapentaenoic acid (n6-DPA), and 4-vinylphenol sulfate) were found to be protective factors for ATB. Secondly, the meta-analysis showed that betaine and 1-LG showed little heterogeneity in different databases, suggesting that ATB was more related to these two metabolites. Finally, this study found that the abnormal pe biosynthesis, metabolism of methionine and betaine, and oxidation pathway of branch chain fatty acids were associated with the pathogenesis of ATB.

In recent years, significant advancements have been made in the field of metabolomics, particularly in the areas of AIDS (Acquired immune deficiency syndrome) [25], exercise [26] and health [27], and has also been applied to the diagnosis of diseases [28]. In addition to diagnosis, these metabolites can also be used as biomarkers to elucidate disease mechanisms, as they can truly reflect the adaptation of bacteria to the metabolome due to growth in vivo and host response to infection and disease [9]. Betaine (or trimethylglycine) is similar to choline (trimethylaminoethanol) but differs in that the carboxylate group at the end of choline is reduced to a hydroxyl group. Betaine can be obtained from dietary sources such as whole grains, beets, and spinach, and can also be synthesized from choline in the liver and kidneys [29, 30]. Firstly, choline is oxidized to betaine aldehyde by mitochondrial choline oxidase (choline dehydrogenase). Then, betaine aldehyde dehydrogenase oxidizes betaine aldehyde to betaine in the mitochondria or cytoplasm. In the liver, betaine acts similarly to the methyl donors of choline, folate, S-adenosylmethionine (SAM), and vitamin B12. Methyl donors are important for liver function, cell replication, and detoxification response. Betaine is also involved in the production of carnitine to protect the kidneys from damage and acts as an osmoprotector in the endomedulla. Betaine is a derivative of glycine that is produced endogenously through choline metabolism, or obtained exogenously through dietary intake. One of its roles is to help regulate homocysteine levels in the blood, which is also an amino acid that, when present at high levels, has been linked to an increased risk of neurovascular diseases, dementia, migraines, developmental disorders, or epilepsy [31–33]. The second is to convert homocysteine to methionine and detoxify it in the human liver and kidney, acting as a methyl donor and having osmoprotective properties [34]. Studies have found that choline, carnitine, and betaine are associated with inflammation and cardiometabolic biomarkers [35]. But they play a good role in chronic diseases such as obesity, diabetes, cancer, and Alzheimer’s disease [36–41]. Somashekar [42] has identified betaine as a potential biomarker for ATB detection, which aligns with the findings of this study. Shin. [43] conducted an in vitro trial using culturedguinea pig lung tissue and observed a significant decrease in betaine metabolism, suggesting the involvement of this metabolite in ATB infection. The fact that betaine was shown to be a risk factor for ATB in this study may be due to its abnormal metabolism leading to a decrease in the production of pro-inflammatory cytokines, which blocks the release of anti-inflammatory cytokines and leads to a decline in human immunity. This makes it difficult for the body to combat the accelerated proliferation of *M*.*tb* and the spread of infectious foci. However, the exact mechanism needs to be further explored with more studies in the future.

Jeffery et al. [44] demonstrated for the first time an association between specific lipid biosynthesis pathways and mycobacterium ATB colony morphology and virulence. Then, Garton et al. [45] found that liposome-positive (fatty triacylglycerol) acidobacter is a biomarker for non-replicating (inert) *M*.*tb* cells in sputum, so identifying this persistent group of *M*.*tb* bacteria in sputum provides an exciting and tractable new opportunity to study chemotherapy response and the spread of ATB. At the same time, it has been found that by increasing levels of monoacylglycerol (1-LG) and/or n-acylethanolamine, inhibitors of endocannabinoid hydrolysis may increase levels of their metabolites. Cannabinoids inhibit the release of TNF-α (Tumor Necrosis Factor), which is a type of tumor necrosis factor that causes the killing and necrosis of tumor cells [46]. The risk of 1-LG is acknowledged, but the role of 1-LG in ATB has not been reported in more literature.

PE is the second most abundant phospholipid in eukaryotic cell membranes and is concentrated in the inner lobules of cell membranes together with phosphatidylserine. In Homo sapiens, PE exists in two biosynthetic pathways [47, 48]. The PE biosynthesis from ethanolamine via the Kennedy pathway for the first. In the beginning, choline is catalyzed to choline phosphate by the cytosol-locating enzyme choline or ethanolamine kinase. Then, the conversion to CDP-choline is catalyzed by choline-phosphate cytotransferase, which is localized to the endoplasmic reticulum. Finally, PE is synthesized by choline/ethanolamine phosphotransferase and cofactors such as magnesium ion or manganese ion. The second pathway is the methylation of phosphatidylserine to synthesize PE. Choline, folate, and methionine metabolism are related because they all affect the production of SAM, which is a common donor of methyl groups in biological reactions [49]. Choline primarily contributes to the formation of cell membranes and neurotransmitters [30].

Cysteine (Cys) and methionine are sulfur-containing amino acids. Cys is synthesized from serine, and in bacteria and plants. Cys is converted from serine (via acetyl serine) by the transfer of hydrogen sulfide. In animals, methionine-derived homocysteine is used as a sulfur source, and its condensation products with serine (cysteine thionine) are converted to cysteine. Inhibition of Cys synthesis leads to reduced fitness and pathogenesis in bacteria [50–53]. Cys acts as a central regulator of REDOX homeostasis through the synthesis of Mycobacterium mercaptan (MSH), the main antioxidant buffer of mycobacterium [54]. In addition, Cys-derived Fe-S clusters [55, 56], sulfatolides (SL-1) [57], and hydrogen sulfide (H_2_S) [58], are essential for the respiration and persistence of *M*.*tb* and for resistance to antibiotics [59, 60]. Higher circulating homocysteine (Hcys) levels are consistently associated with HIV-infected patients [61]. Bandyopadhyay [62] theorizes that cysteine beta synthase may contribute to the survival of *M*. *tb* during HIV/TB co-infection. Methionine is an essential amino acid that cannot be synthesized by animals. In bacteria and plants, methionine is synthesized from aspartate. SAM is a methyl donor synthesized from methionine and Adenosine Triphosphate (ATP) and acts as a methyl donor in many important transfer reactions, including DNA methylation. SAM may also be used to regenerate methionine in the methionine recycling pathway. Methionine is involved in countless cellular functions, and methionine is essential for immunity against tumors. Methionine metabolism is involved in many cellular functions, including methylation reactions, redox maintenance, and folate metabolism. Acetylation and methylation are important for controlling antimicrobial mechanisms within macrophages (MΦ) during intracellular infections such as ATB [63, 64]. The inactivation of cysteine and methionine biosynthesis in *M*.*tb* significantly reduces the persistence and virulence of the chronic infection phase in mouse models [65].

In addition, patients with obesity and insulin resistance are often accompanied by abnormal increases in branched-chain amino acids (BCAAs) and their metabolites. Metabolic disorders of BCAAs are strongly associated with the development of obesity and insulin resistance. In addition, levels of monomethyl-branched-chain fatty acids (mmBCFAs) have also been linked to the development of metabolic syndrome and cancer. However, there are few studies on mmBCFAs at present, and the specific mechanism of mmBCFAs’ action in disease is not fully understood, further studies are needed to reveal its role in disease development. Only Kniazeva [66, 67] studies have mentioned that lipids containing mmBCfAs play a key role in the postembryonic growth, neuronal development, and foraging behavior of Caenorhabditis elegans.

It is worth mentioning that the ATB bacterium has a special cell wall structure containing branched-chain fatty acids (mycolic acid), which play a key role in the formation and stability of the cell wall. The ATB bacterium maintains the integrity of its cell wall by synthesizing branched fatty acids, thereby protecting itself from attack by the host immune system. The metabolism of branched-chain fatty acids is closely related to the synthesis and assembly of cell wall polysaccharides, and together these molecules make up an important part of the cell wall of the ATB bacterium. Therefore, the metabolism of branch-chain fatty acids interacts with the synthesis of cell wall polysaccharides to affect the growth, reproduction, and pathogenicity of ATB bacteria.

In conclusion, in the future, when developing and diagnosing ATB drugs, it may be considered to start from the above metabolites and monitor them through multiple stages using in vitro and in vivo models to evaluate their role in the progression of ATB. At the same time, although this study only focused on the significance of PE biosynthesis, methionine metabolism, betaine metabolism, and branched-chain fatty acid metabolism pathways, the role of these pathways in the pathogenesis of ATB should be further explored in the process of drug development.

However, there may be several limitations to this study. Firstly, in the current environment, it was inevitable to encounter the limitation of unavailability of individual behavioral and biological characteristics due to the utilization of a pooled dataset. Secondly, there is a possibility that certain metabolites associated with ATB might have been excluded due to the pleiotropic level limitation of instrumental variables or limitations in the sample size. Lastly, some significant metabolites and pathways were omitted as they lacked proper naming or annotation in pathway databases. Therefore, further comprehensive exploration will be necessary in future studies.

## Conclusion

Through a causal analysis based on a large European sample, we found that betaine and 1-LG may be biomarkers or auxiliary diagnostic tools for ATB. The findings suggest that these two human serum metabolites may lead to new guidance for medical practice in the early diagnosis and surveillance of ATB. In addition, by interfering with PE synthesis, methionine metabolism, betaine metabolism branch chain fatty acid metabolism, and other pathways, it is helpful to develop new anti-tuberculosis drugs and explore the virulence or pathogenesis of ATB at a deeper level, providing an effective reference for future studies.

## Supporting information

S1 Table STROBE-MR checklist of recommended items to address in reports of mendelian randomization studies. (CSV)

S2 Table The super-pathway of metabolites selected by IVs. (CSV)

S3 Table The causal relationships between 57 metabolites and the risk of ATB are estimated by three MR models, which also conduct tests for heterogeneity and horizontal pleiotropy. (CSV)

S1 Dataset (XLSX)

## Abbreviation

AIDS: Acquired immune deficiency syndrome
ATB: Active tuberculosis
ATP: Adenosine Triphosphate
BCAAs: branched-chain amino acids
KEGG: Kyoto Encyclopedia of Genes and Genomes
CI: confidence interval
Cys: Cysteine
GWAS: Genome Wide Association Study
HIV: Human Immunodeficiency Virus
IEU: Integrative Epidemiology Unit
IVs: Instumental variables
IVW: Inverse variance weighting
Kb: Kilobase
KEGG: Kyoto Encyclopedia of Genes and Genomes
LD: Linkage disequilibrium
LG: 1-linoleoylglycerol (1-monolinolein)
M.tb: Mycobacterium tuberculosis
mmBCFAs: monomethyl-branched-chain fatty acids
MR: Mendelian randomization
OR: Odds ratio
PE: phosphatidylethanolamine
SAM: S-adenosylmethionine
SMPDB: Small Molecule Pathway Database
SNPs: Single nucleotide polymorphisms
TB: Tuberculosis
TBSA: Tuberculous stearic acid
TNF-α: Tumor Necrosis Factor α
WM: Weighted median
